# Wnt/pathway, APP processing and telomere shortening in Down syndrome adults

**DOI:** 10.1002/alz.092775

**Published:** 2025-01-09

**Authors:** Augusto Magno Tranquezi Cordeiro, Jessyka Maria de França Bram, Leda Leme Talib, Orestes Vicente Forlenza, Lillian Fernandes

**Affiliations:** ^1^ Instituto de Psiquiatria do Hospital das Clínicas da Faculdade de Medicina da Universidade de São Paulo, São Paulo, São Paulo Brazil; ^2^ Laboratory of Neurosciences, Institute of Psychiatry, Faculty of Medicine, University of São Paulo, Sao Paulo, Sao Paulo Brazil; ^3^ Memory Café Brasil, São Paulo Brazil; ^4^ Laboratório de Neurociências ‐ LIM 27, Sao Paulo Brazil; ^5^ University of São Paulo Medical School, São Paulo Brazil; ^6^ Faculdade de Medicina da Universidade de São Paulo, São Paulo, São Paulo Brazil; ^7^ Laboratory of Neuroscience (LIM‐27), Department and Institute of Psychiatry, Faculty of Medicine, University of São Paulo, São Paulo, São Paulo Brazil; ^8^ University of São Paulo Universidade de São Paulo, São Paulo Brazil

## Abstract

**Background:**

The Down Syndrome (DS), also referred to as trisomy of chromosome 21, is a prevalent cause of intellectual disability and also contributes to the acceleration of aging, among other developmental and health concerns. Certain pathological characteristics shared by DS and Alzheimer’s Disease (AD) indicate similar commonalities.

This study aims to unravel the relationship between the canonical Wnt/pathway, the amyloid precursor protein processing, the telomere shortening in DS individuals. The telomere is a structure found at the end of the chromosomes that protects against degradation during cell division. The shortening of the telomere length is associated with the risk of AD. β‐catenin is an important factor in the canonical Wnt pathway. The activation of this pathway can protect against neurotoxic effects caused by accumulation of β‐amyloid peptide and the phosphorylation of Tau protein. The canonical Wnt activation is related with the activation of subunit Tert, also known as telomerase. Telomerase is an enzyme responsible for the maintenance of telomere length.

**Methods:**

The biomarkers were quantified in the plasma and platelets of adults and elderly with DS (n = 55) and adults and elderly without DS (n = 80). We utilized western blot, for APP fragments (110 kDa and 130 kDa), Luminex xMAP, for AB40 fragment. The DNA was extracted from leukocytes and measured using qPCR method using 36B4 as single copy gene. Telomere length was calculated using the delta Ct value.

**Results:**

We found increased levels of Aβ40 and in 110 kDa, 130 kDa APP fragments of Down syndrome individuals. β‐catenin was also increased in DS group when compared with non DS group. The telomere length was decrease in DS group when compared with non DS group.

**Conclusion:**

The increase in Aβ40 and 110 kDa, 130 kDa fragments, demonstrated an imbalance in the APP processing, that could lead to the Aβ aggregation observed in DS individuals. The increase in the β‐catenin expression could be a compensatory mechanism for the telomere shortening in DS group.

